# How has mass drug administration with dihydroartemisinin-piperaquine impacted molecular markers of drug resistance? A systematic review

**DOI:** 10.1186/s12936-022-04181-y

**Published:** 2022-06-11

**Authors:** Sophie Moss, Emilia Mańko, Sanjeev Krishna, Susana Campino, Taane G. Clark, Anna Last

**Affiliations:** 1 Faculty of Infectious and Tropical Diseases, London School of Hygiene & Tropical Medicine, London, UK; 2 Institute of Infection and Immunity, St George’s University of London, London, UK; 3 Faculty of Epidemiology and Population Health, London School of Hygiene & Tropical Medicine, London, UK

**Keywords:** Antimalarial resistance, Dihydroartemisinin-piperaquine, Mass drug administration, Molecular markers

## Abstract

The World Health Organization (WHO) recommends surveillance of molecular markers of resistance to anti-malarial drugs. This is particularly important in the case of mass drug administration (MDA), which is endorsed by the WHO in some settings to combat malaria. Dihydroartemisinin-piperaquine (DHA-PPQ) is an artemisinin-based combination therapy which has been used in MDA. This review analyses the impact of MDA with DHA-PPQ on the evolution of molecular markers of drug resistance. The review is split into two parts. Section I reviews the current evidence for different molecular markers of resistance to DHA-PPQ. This includes an overview of the prevalence of these molecular markers in *Plasmodium falciparum* Whole Genome Sequence data from the MalariaGEN Pf3k project. Section II is a systematic literature review of the impact that MDA with DHA-PPQ has had on the evolution of molecular markers of resistance. This systematic review followed PRISMA guidelines. This review found that despite being a recognised surveillance tool by the WHO, the surveillance of molecular markers of resistance following MDA with DHA-PPQ was not commonly performed. Of the total 96 papers screened for eligibility in this review, only 20 analysed molecular markers of drug resistance. The molecular markers published were also not standardized. Overall, this warrants greater reporting of molecular marker prevalence following MDA implementation. This should include putative *pfcrt* mutations which have been found to convey resistance to DHA-PPQ in vitro*.*

## Background

The evolution of anti-malarial drug resistance presents an alarming threat to eliminating malaria; a disease which causes over 500,000 deaths every year [1]. Malaria is caused by the protozoan parasite *Plasmodium*, with most fatal cases caused by *Plasmodium falciparum* [1]*.* The role of Mass Drug Administration (MDA) in the evolution of anti-malarial resistance is not well understood. MDA is defined by the WHO as the mass treatment of all, or a section of, the population, whether or not symptoms are present [2]. Historical MDA practices, such as the addition of the anti-malarial chloroquine to table salt, have been correlated with a subsequent rise in chloroquine resistance [3]. However, there is a lack of evidence linking more recent use of MDA with the evolution of anti-malarial resistance in *P. falciparum*. This review is focused on dihydroartemisinin-piperaquine (DHA-PPQ); an increasingly used artemisinin-combination therapy (ACT) in MDA for malaria. The first section of this review details current evidence on the molecular mechanisms behind resistance of *P. falciparum* to DHA-PPQ. This section also includes a comprehensive overview of the prevalence of molecular markers associated with DHA-PPQ resistance globally, using Whole Genome Sequence data from the MalariaGEN Pf3k project. The second section of this review systematically evaluates the impact of MDA with DHA-PPQ on the evolution of anti-malarial resistance. The authors systematically reviewed the evidence from the available literature reporting molecular markers of anti-malarial resistance following MDA with DHA-PPQ.

### Section I: the evolution of drug resistance

Anti-malarial drugs can be grouped into broad classes (Table 1). Widespread resistance to monotherapies led the World Health Organization (WHO) to recommend artemisinin-based combination therapy (ACT) as first-line treatment in all malaria endemic countries for uncomplicated malaria, with artesunate recommended to treat severe cases [4]. ACT includes a combination therapy of an artemisinin derivative (artesunate, artemether or dihydroartemisinin) with a partner drug (either lumefantrine, amodiaquine, piperaquine, mefloquine or sulfadoxine-pyrimethamine) [4]. The most common artemisinin-based combinations used in Africa are artemether-lumefantrine (AL), artesunate-amodiaquine (AS-AQ) and dihydroartemisinin-piperaquine DHA-PPQ [4]. All currently have a high clinical efficacy in Africa, achieving 98%, 98.4% and 99.4%, respectively [4].

**Table 1:** Anti-malarial drugs can be broadly grouped into different classes [27]

Class	Antimalarial drugs
4-Aminoquinolines	Chloroquine, amodiaquine, piperaquine
8-Aminoquinolines	Primaquine, tafenoquine
Antifolates	Pyrimethamine, sulphadoxine, proguanil
Amino-alcohols	Lumefantrine, mefloquine, quinine
Mannich base	Pyronaridine, naphthoquinone, atovaquone
Sesquiterpenes	Artemisinin and its derivatives; dihydroartemisinin, artemether, artesunate, arteether, artemisone
Antibiotics	Doxycycline, clindamycin

**Fig. 1:**
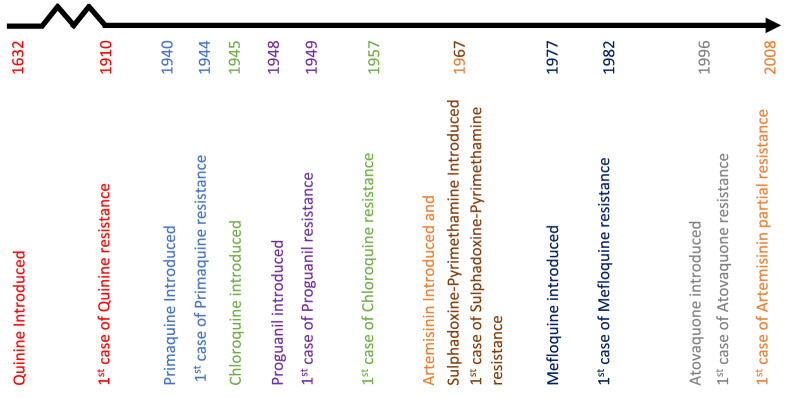
A timeline of the evolution of resistance to anti-malarial drugs. Each drug has been given a different colour for ease of timeline interpretation [7–10]

Resistance to many anti-malarial drugs has now evolved, but the speed at which resistance has emerged has differed depending on the drug (Fig. 1). Understanding the molecular mechanisms behind the evolution of resistance in these different drugs is crucial in understanding why resistance has evolved at different rates. Furthermore, the methods used for detecting and classifying anti-malarial resistance have changed over time [5]. In addition to in vivo*, *in vitro and ex vivo methods*,* molecular methods for detecting resistance have now been developed. This includes identifying genomic polymorphisms in the malaria parasite genome which are associated with resistance to anti-malarial drugs [5]. Historically, anti-malarial drug resistance has often spread from Southeast Asia to Africa, so monitoring molecular markers of resistance in different continents may enable the scientific community to pre-empt the spread of drug resistant malaria in Africa [6], depending on the mechanisms of resistance.

### Dihydroartemisinin-piperaquine: how does it work?

**Fig. 2:**
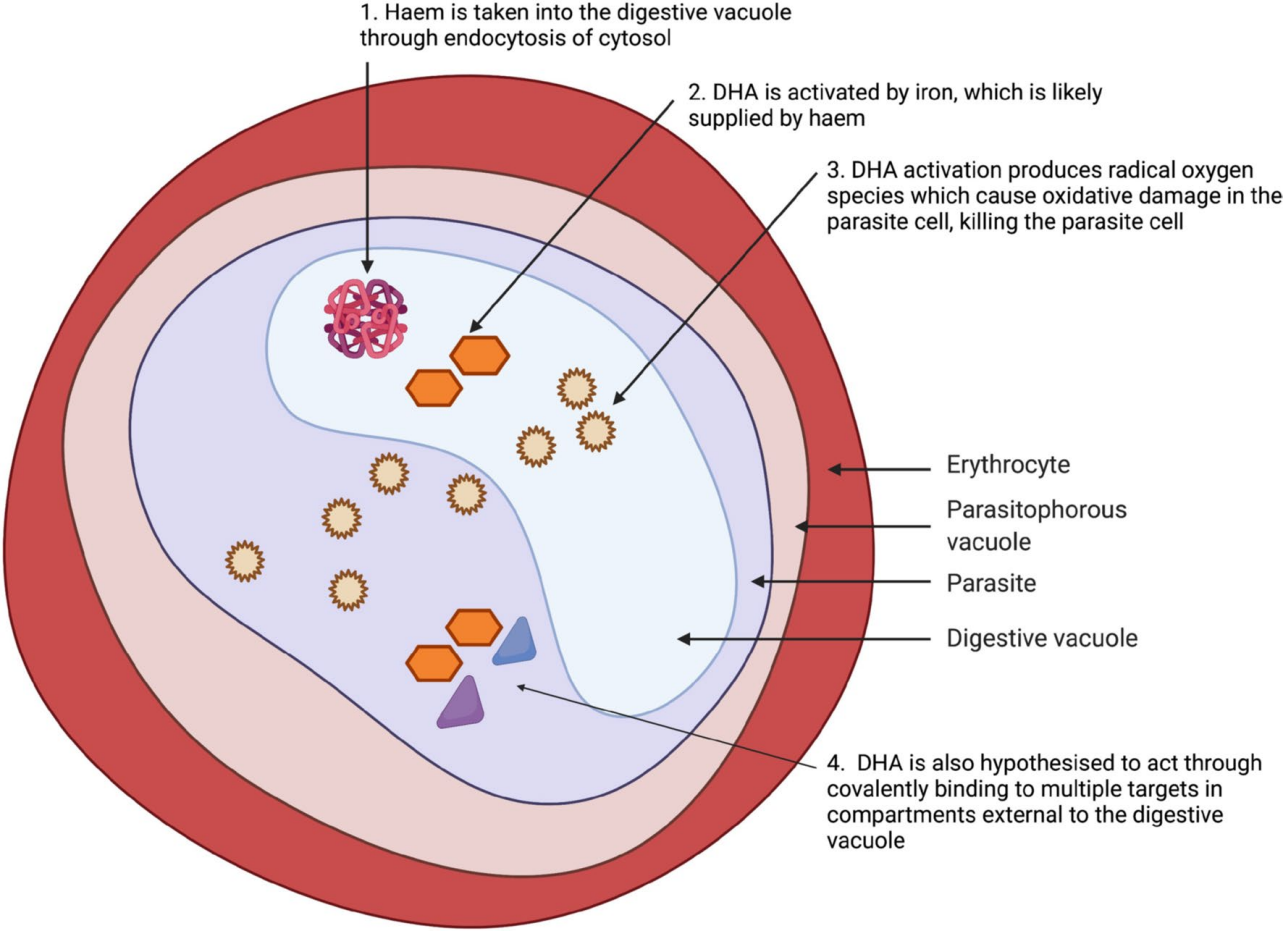
A diagram illustrating how DHA is predicted to attack the parasite cell. Created with BioRender.com

Dihydroartemisinin-piperaquine (DHA-PPQ) is an artemisinin-based combination composed of fast acting dihydroartemisinin, and slow acting piperaquine. Dihydroartemisinin (DHA) is a synthetic derivative of artemisinin, which is a sesquiterpene lactone first extracted from the plant *Artemisia annua* in 1972 [11, 12]. DHA is activated by iron, which is likely supplied by haem [13] which is taken into the parasite digestive vacuole (DV) through endocytosis of cytosol [14]. The most popular hypothesis for DHA’s mechanism of action is that, once activated by haem, DHA produces radical oxygen species which cause oxidative damage in the parasite cell, killing the parasite [13, 15]. DHA is also hypothesized to act through the formation of covalent bonds with multiple targets in compartments external to the DV [16]. These mechanisms of action are illustrated in Fig. 2. DHA acts quickly and has a short half-life of approximately 0.85–2 h in adults [17–19]. To clear any residual parasites following the rapid action of DHA, it is paired in combination therapy with the long-acting partner drug, piperaquine [20].

**Fig. 3:**
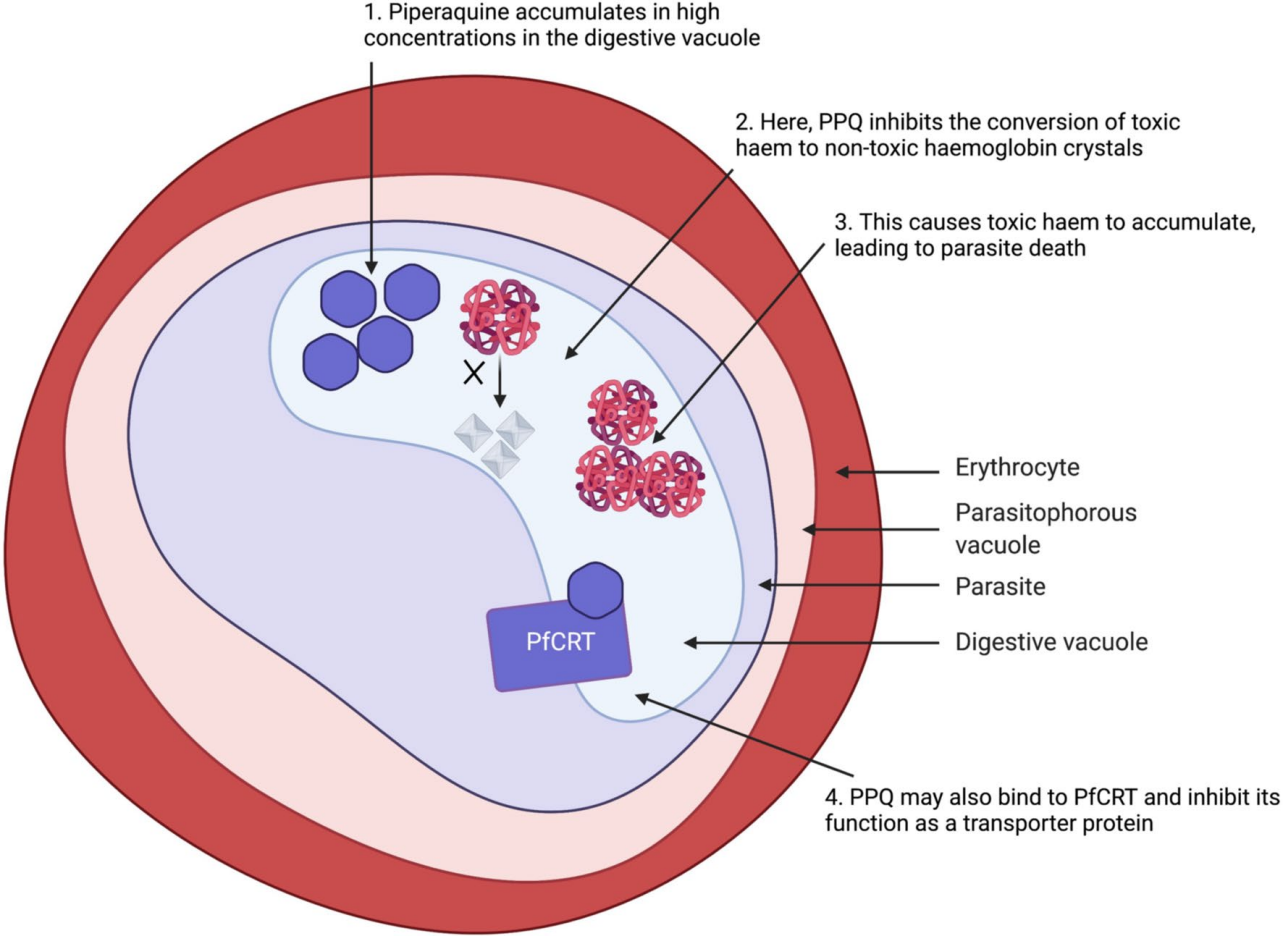
A diagram illustrating how PPQ is predicted to attack the parasite cell. Created in Biorender.com

Piperaquine (PPQ) was first introduced as a monotherapy in the 1960s, and later as the partner drug in DHA-PPQ combination therapy [20]. Piperaquine is thought to act by accumulating in high concentrations in the parasite’s digestive vacuole. Here, it inhibits the conversion of toxic haem to non-toxic haemozoin crystals during parasite haemoglobin digestion, which is an essential metabolic process for the parasite. Inhibiting the conversion of haem to haemozoin results in high concentrations of toxic haem accumulating in the digestive vacuole, leading to parasite death [21, 22]. Furthermore, in vitro studies have demonstrated that *P. falciparum* exposed to PPQ accumulate more undigested haemoglobin, suggesting that PPQ decreases the rate of haemoglobin digestion, possibly killing the parasite through ‘starvation’ [23]. There is also evidence suggesting that PPQ binds directly to the *P. falciparum* chloroquine resistance transporter, PfCRT [24], where it may inhibit PfCRT’s usual function as a transporter protein. These mechanisms of action are illustrated in Fig. 3.

### Dihydroartemisinin-piperaquine: what are the resistance mechanisms?

Resistance to DHA-PPQ has emerged in Southeast Asia [25, 26] but the molecular mechanism of resistance is not fully understood. Molecular markers for partial resistance to DHA include single nucleotide polymorphisms (SNPs) on the *pfk13 Plasmodium* gene, including the mutations F446I, N458Y, M476I, Y493H, R539T, Y543T, P553L, R561H, P574L and C580Y, which have been validated by the WHO [27]. Resistance to the partner drug PPQ is less well understood. Resistance to PPQ is associated with gene duplication of the plasmepsins *pfpm2* and *pfpm3* [28–32], and inactivation of either of these genes increases sensitivity to PPQ [33]. Plasmepsins II and III are proteases which work in a complex with other proteases in the digestive vacuole (DV) to digest haemoglobin and produce essential amino acids for the parasite [34]. Plasmepsin duplications may facilitate resistance to PPQ by increasing the rate of haemoglobin digestion. This may counteract the inhibitory effects of PPQ on haemoglobin digestion. Of note, resistance to PPQ has also been shown without duplication of *pfpm2* [35–37] and there is some evidence that increased expression of *pfpm2* and *pfpm3* does not alter PPQ susceptibility [38]. Therefore, although PPQ resistance is correlated with *pfpm2* and *pfpm3* duplication, this is unlikely to be the sole mechanism of PPQ resistance. Considering this, plasmepsin copy number should not be used as the only indicator for surveying PPQ resistance. As with other anti-malarial drugs, such as mefloquine [39], all cases of resistance cannot usually be explained completely by one specific genetic polymorphism.

Resistance to DHA-PPQ has been associated with other genetic polymorphisms, including on the *pfexo* and *pfcrt* genes*. Plasmodium falciparum* Exonuclease (*pfexo*) is a putative exonuclease encoding gene. The E415G polymorphism on *pfexo* is strongly linked to increased copy number of *pfpm2* and *pfpm3* and has been correlated with treatment failure of DHA-PPQ in Cambodia [29, 40]. However, the functional role of this protein is uncertain.

Multiple polymorphisms in *pfcrt* are correlated with PPQ resistant phenotypes. The mutations T93S, H97Y, F145I and I218P have been associated with DHA-PPQ resistance in Cambodian isolates. Furthermore, in vitro studies have found that H97Y, F145I, M343L, G353V [21] and C101F [22] cause the Dd2 (“Indo-China”) laboratory strain to be PPQ resistant and CQ sensitive*. *In vitro data has shown that F145I and C350R can mediate efflux of PPQ from the DV in the 7G8 (“Brazil”) laboratory strain at the same time as reducing CQ transport, suggesting that PPQ resistance may arise through efflux of PPQ from the digestive vacuole via PfCRT, in a similar mechanism to CQ resistance [24]. The mutations T93S and I218P have been shown to confer resistance to PPQ without the presence of *pfpm2* duplications [35]. Furthermore, T93S, H97Y, F145I and I128F each conferred resistance to PPQ on a background of *pfexo* E145G, but again without *pfpm2* duplication [36]. These data further show that plasmepsin duplications are not required for PPQ resistance. Many of these *pfcrt* mutations also resulted in a swollen digestive vacuole [21, 22, 36, 41]. This vacuole swelling indicates that the PfCRT protein has a role in maintaining vacuole morphology, and that mutations in *pfcrt* disrupt this function [41]. The structure of PfCRT has recently been elucidated [24], showing that *pfcrt* mutations associated with PPQ resistance are at moderately conserved sites in selected helices of the protein, including T93S, H97Y and C101F. This work has highlighted a number of other amino acid sites with similar properties, which may be under similar selection pressures from PPQ and may be useful for future PPQ resistance surveillance [24].

One hypothesis for the mechanism of PPQ resistance is that *pfcrt* mutations enable PfCRT to transport PPQ out of the digestive vacuole, away from its putative site of action, similarly to CQ resistance. However, some of these *pfcrt* mutations conveyed PPQ resistance without changing the rate of PPQ transport out of the digestive vacuole [21]. A competing hypothesis is that PPQ binds to PfCRT as part of its mode of action, disrupting its role as a transporter and DV morphology regulator, causing parasite death. These *pfcrt* mutations may inhibit PPQ from binding, causing PPQ resistance, whilst simultaneously removing the transporter’s ability to transport CQ, leading to CQ susceptibility [22].

Polymorphisms in *pfmdr1* have been associated with PPQ sensitivity. Conrad et al. found that DHA-PPQ treatment selected for the *pfmdr1* haplotype 86Y/Y184/Y1246. Interestingly, treatment with Artemether-Lumefantrine (AL) selected for opposite alleles; N86/184F/D1246 [42]. These opposing selection pressures suggest that DHA-PPQ may be a good choice of partner drug in areas where AL was previously used. Furthermore, increased *pfmdr1* copy number has been associated with enhanced sensitivity to piperaquine [29, 43]. Veiga et al. hypothesized that increased *pfmdr1* copy number is associated with enhanced accumulation of PPQ in the DV, leading to increased sensitisation to PPQ [43].

In summary, PPQ is likely to kill parasites by disrupting haemoglobin digestion and may also act by binding to PfCRT, disrupting its role as a transporter. *Pfpm2* and *pfpm3* duplications correlate with PPQ resistance, but duplication is not essential for resistance. Therefore, plasmepsin copy number should not be used as a sole indicator of PPQ resistance. Additionally, some polymorphisms in *pfcrt* can confer resistance to PPQ in Dd2 parasites and the E415G *pfexo* mutation has been correlated with DHA-PPQ resistance in Cambodian isolates. Finally, increased *pfmdr1* copy number has been associated with enhanced sensitivity to PPQ.

### How prevalent are these putative PPQ-resistance conferring mutations?

**Fig. 4:**
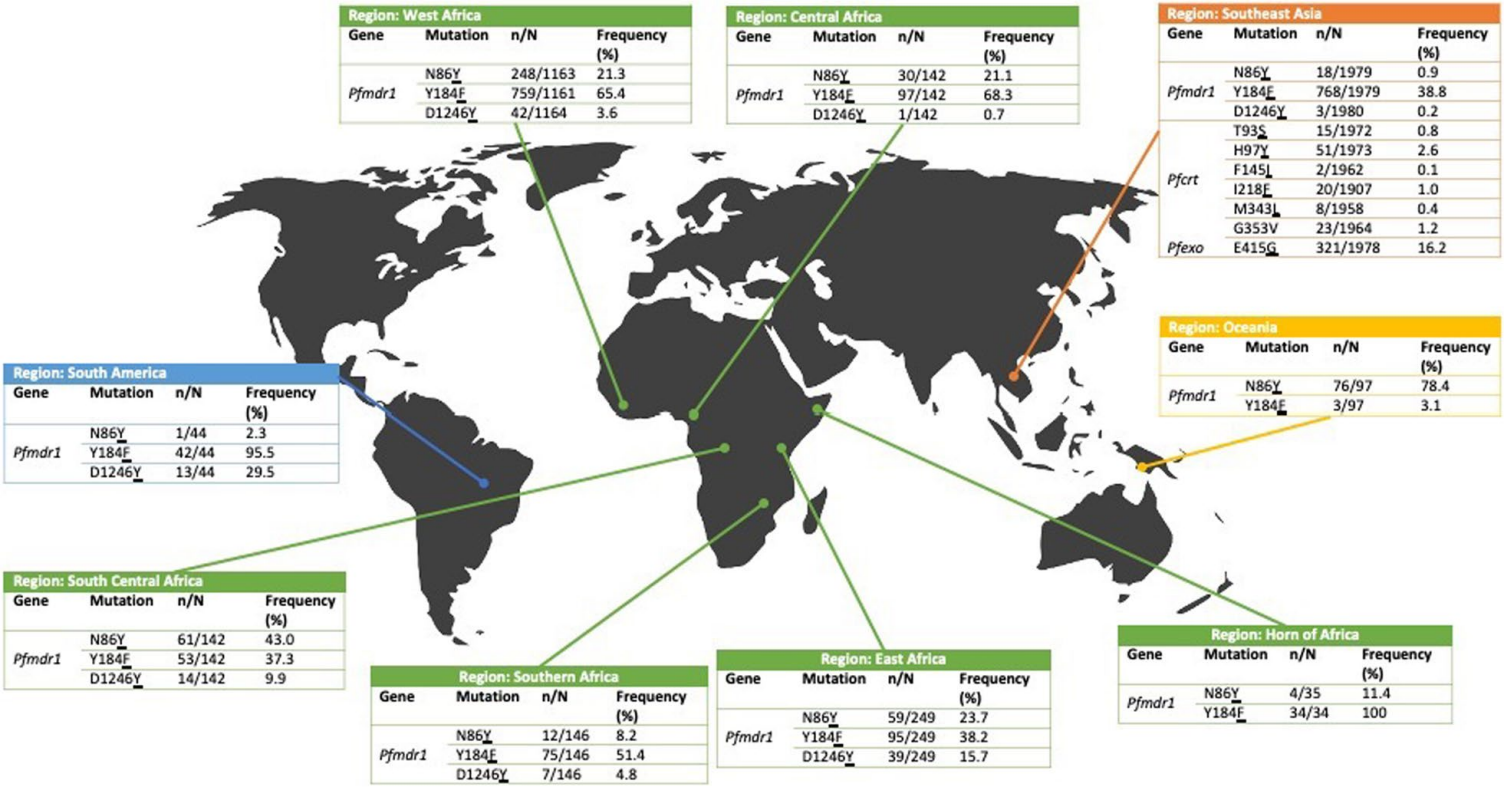
A diagram showing the global frequencies of mutations in the *pfmdr1, pfcrt and pfexo* genes, which are potential markers of DHA-PPQ resistance. These frequencies were calculated using whole genome sequence data from recent studies [44–46]. n is the number of samples containing a mutant allele, and N is the total number of successfully sequenced samples. Total sample size = 4001

As part of this review, whole genome sequence data was used to analyse the prevalence of the above-mentioned SNPs in *P. falciparum* samples from studies worldwide [44–46] (n = 4001) (Fig. 4). For frequency calculations, the authors considered isolates with monoclonal infections based on the Fws metric. The *pfmdr1* N86**Y** mutation was found at a prevalence of between 21.1% and 23.7% in samples from Central, West and East Africa, and a lower prevalence of 11.4% in the Horn of Africa and 8.2% in Southern Africa. A higher prevalence was found in Southern Central Africa, at 43%. The prevalence of N86**Y** was also higher in Oceania, at 78.4%. Whereas, the prevalence was lower in samples from South America, at 2.3% and Southeast Asia, at 0.9%. The *pfmdr1* Y184**F** mutation was found at a prevalence between 37.3% and 51.4% in samples from South Central Africa, East Africa, Southeast Asia and Southern Africa. The prevalence was 65.4% in West Africa and 68.3% in Central Africa. This mutation was found at a much higher prevalence of 95.5% in South America and in the Horn of Africa, where it was found to be 100%. In comparison, the prevalence of Y184**F** was very low in Oceania, at a prevalence of 3.1%. The D1246**Y** mutation was not found in samples from Oceania or the Horn of Africa, and ranged from a very low prevalence of 0.2% in Southeast Asia, to 29.5% in South America. *Pfcrt* mutations of interest, including T93**S**, H97**Y**, F145**I**, I218**F**, M343**I** and G353**V**, were only present in samples from Southeast Asia, with a mutation prevalence of 0% in the other global regions. The prevalence of these muations was low, with T93**S**, F145**I**, M343**L** in under 1% of the samples analysed. I218**F** had a prevalence of 1% and G353**V** had a prevalence of 1.2%, while H97**Y** had a prevalence of 2.6%. The *pfexo* mutation E415**G** was only found in samples from Southeast Asia, at a prevalence of 16.2%.

## Section II: how has mass drug administration with DHA-PPQ impacted molecular markers of resistance?

Monitoring the prevalence of molecular markers associated with DHA-PPQ resistance enables widespread surveillance of *P. falciparum* markers in populations undergoing mass drug administration (MDA). This molecular surveillance can then be used to inform treatment policy specific to different populations. This review has investigated the impact of MDA with DHA-PPQ on the evolution of molecular markers associated with anti-malarial resistance.

## Methodology

**Fig. 5:**
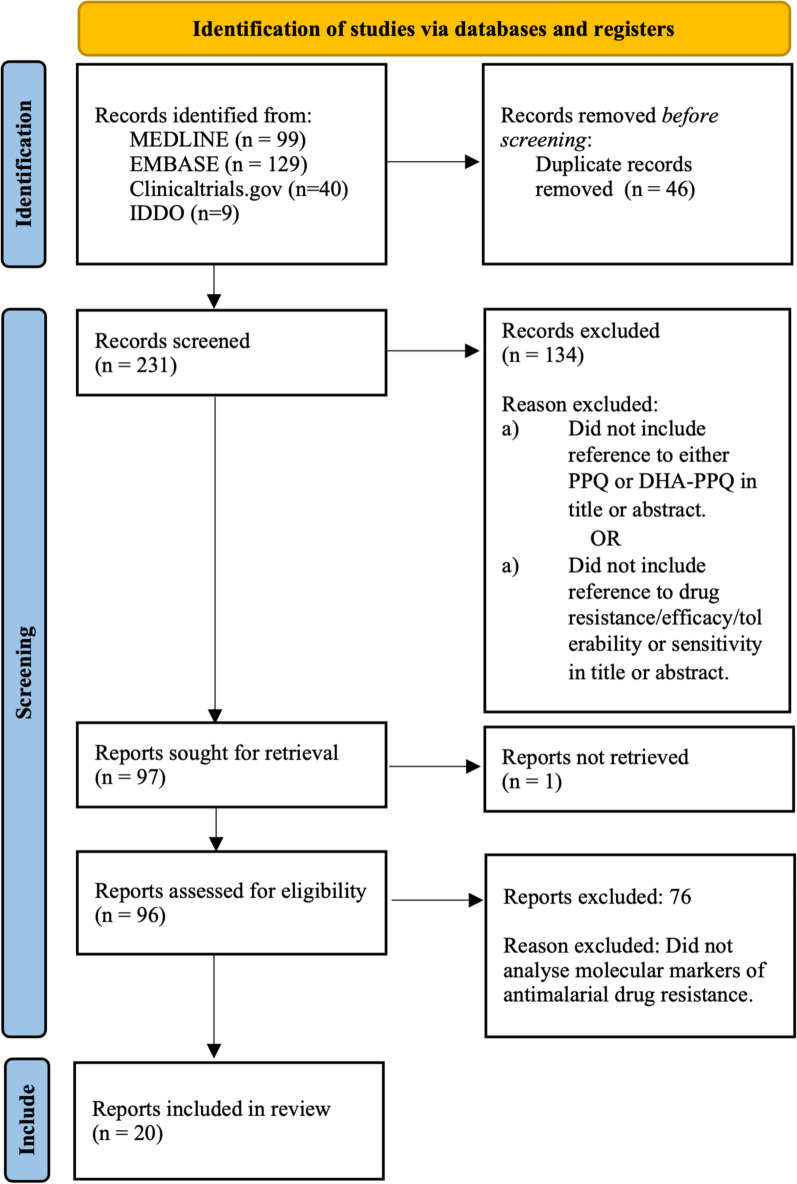
A flowchart demonstrating how studies were idendified for this review following PRISMA guidelines

This review included relevant studies from clinicaltrails.gov, EMBASE, MEDLINE and the Infectious Diseases Data Observatory (IDDO). Searches were dated back to 2011, when DHA-PPQ was first approved as the ACT Eurartesim^®^, by the European Medicines Agency. A detailed search strategy and methodology can be found in Appendix 1, which follows PRISMA guidelines. In brief, MDA studies were included from www.clinicaltrials.gov which were completed, with results, which used DHA-PPQ or PPQ as an intervention. Associated publications with reference to molecular markers of resistance were included in this review. Two further publications were included from IDDO, following search terms ‘malaria’ and ‘piperaquine’. Search terms for malaria, mass drug administration, anti-malarial resistance and PPQ or DHA-PPQ were used to extract publications from EMBASE and MEDLINE. These were then filtered for publications which included analysis of molecular markers of resistance. The methodology flowchart can be seen in Fig. 5.

## Results

A total of 20 studies passed the screening criteria and were included for analysis in this systematic review. Each study was analysed to understand the reported impact of treatment with DHA-PPQ or PPQ on molecular markers associated with resistance to DHA-PPQ. Molecular marker data extracted from these studies included *pfpm2* copy number, *pfexo* E415**G**, *pfcrt* mutations*, pfmdr* mutations and copy number variations*,* and artemisinin-resistance associated mutations. Of the 20 studies reviewed, 7 included analysis of *pfpm2* copy number, 2 included analysis of *pfexo* E415**G**, 9 included analysis of mutations in *pfcrt,* 13 included analysis of mutations and/or copy number variation in *pfmdr1* and 11 included analysis of mutations associated with reduced artemisinin-sensitivity. 16 of the studies were associated with clinical trials which included the use of DHA-PPQ. 2 used piperaquine phosphate and 1 used arteminisinin-piperaquine. 14 studies were associated with MDA trials, 7 of which were associated with IPTp, which is a form of targeted MDA. 5 studies were associated with clinical trials for treatment of confirmed malaria, but were retained in this review to provide the breadth of genomic data available related to DHA-PPQ use. A summary of the relevant molecular markers reported in each study can be seen in Table 2, sorted by molecular marker of interest. One report analysed molecular markers in the *pfdhfr and pfdhps* genes associated with SP resistance, but did not analyse polymorphisms associated with resistance to DHA-PPQ in *pfpm2, pfmdr1, pfcrt or pfkelch13* [49]*.* Therefore, this study is not included in Table 2.

**Table 2:** A summary of the papers which were analysed in this systematic literature review, detailing which molecular markers were detected following treatment, and any changes in the frequency of molecular markers investigated

Molecular marker associated with DHA-PPQ resistance	Associated trial(s)	Intervention	Publication	Impact of intervention on molecular marker
*Pfpm2 copy number*	Samples from multiple trials and epidemiological studies conducted between 1st Jan 2007 and 31st December 2018 were analysed by Imwong et al. (2020). Therapeutic efficacy studies of artesunate [50] and DHA-PPQ in Yunnan Province of China, from 2009 to 2012	Multiple trials and epidemiological studies, including MDA with DHA-PPQ in Kayin State, Myanmar	Imwong et al. (2020) [51] Molecular epidemiology of resistance to antimalarial drugs in the Greater Mekong subregion: an observational study	This study found that between Jan 1 2007 and Dec 31 2018, *pfpm2* amplification was more frequent in the eastern Grater Mekong subregion than in Myanmar. Regarding mass drug administration, there was no evidence of selection for increased *pfpm2* copy number following MDA with DHA-PPQ in Myanmar and Cambodia
	NCT02914145 Mass Drug Administration of Monthly DHA-PQP to Accelerate Towards Malaria Elimination in Magude District, Southern Mozambique	Mass Drug Administration with DHA-PPQ. Two monthly rounds of MDA with DHA-PPQ for two consecutive years in Magude, Southern Mozambique	Gupta et al. (2020) [52] Effect of mass dihydroartemisinin–piperaquine administration in southern Mozambique on the carriage of molecular markers of antimalarial resistance	There was no statistically significant difference in the proportion of isolates with multicopy *pfpm2* when comparing samples collected pre and post MDA
	NCT02282293 Reducing the Burden of Malaria in HIV-Infected Pregnant Women and Their HIV-Exposed Children (PROMOTE Birth Cohort 2)	Randomized controlled trial among 300 pregnant women in Uganda. Participants were randomized to receive either: (i) SP (500 mg Sulphadoxine and 25 mg pyrimethamine) every 8 weeks (ii) DHA-PPQ (40 mg dihydroartemisinin and 320 mg PQ) every 8 weeks or (iii) DHA-PPQ every 4 weeks	Conrad et al. (2017) [53] Impact of Intermittent Preventive Treatment During Pregnancy on *Plasmodium falciparum* Drug Resistance–Mediating Polymorphisms in Uganda	This study found a modest increase in *pfpm2* copy number in 1 of 18 samples from patients receiving DHA-PPQ IPTp
	NCT01872702 Targeted Chemo-elimination (TCE) to Eradicate Malaria in Areas of Suspected or Proven Artemisinin Resistance in Southeast Asia and South Asia	Active comparator: Three monthly rounds of DHA-PPQ and low dose primaquine Placebo comparator: No intervention used Two villages randomly allocated to intervention at each of 4 sites, and two villages randomly allocated to control (no intervention). 500 people per village	von Seidlein et al., (2019) [54] The impact of targeted malaria elimination with mass drug administrations on *falciparum* malaria in Southeast Asia: A cluster randomized trial	This study analysed blood specimens from before MDA initiation. They found that 4% of the individuals sampled (10 of 269) had *pfkelch13* C580Y and multiple copies of *pfpm2/3.* This study did not measure the difference in prevalence of markers pre and post MDA
			Landier et al. (2017) [55] Safety and effectiveness of mass drug administration to accelerate elimination of artemisinin-resistant *falciparum* malaria: a pilot trial in four villages of Eastern Myanmar	This study found no amplification of *pfpm2* copy number in the 69 samples that they analysed, which were 53 collected before the MDA and 16 collected afterwards
	NCT02083380 Randomizedd Phase IIb Study of Efficacy, Safety, Tolerability & Pharmacokinetics of a Single Dose Regimen of Artefenomel (OZ439) in Loose Combination With Piperaquine in Adults and Children With Uncomplicated *Plasmodium falciparum* Malaria	Experimental: A) Artefenomel 800 mg and Piperaquine Phosphate 640 mg Experimental: B) Artefenomel 800 mg and Piperaquine Phosphate 960 mg Experimental: C) Artefenomel 800 mg and Piperaquine Phosphate 1440 mg	Leroy et al. (2019) [56] African isolates show a high proportion of multiple copies of the *Plasmodium falciparum* plasmepsin-2 gene, a piperaquine resistance marker	This study analysed *pfpm2* copy number in samples collected from patients before treatment. They found a higher proportion of multicopy parasites in African samples compared to Asian samples. Parasites with multicopy *pfpm2* and single copy *pfmdr1* (hypothesized to favour PPQ resistance) were found at similar prevalence in Asian and African samples
	Samples from multiple trials and epidemiological studies were analysed by Imwong et al. (2017). Those that were trials that included PPQ or DHA-PPQ were NCT02453308	Longitudinal observational study between 1st January 2008 and 31st December 2015	Imwong et al. (2017) [57] The spread of artemisinin-resistant *Plasmodium falciparum* in the Greater Mekong subregion: a molecular epidemiology observational study	This study found *pfpm2* amplification in isolates collected between 1^st^ January and 31^st^ December 2008, in western Cambodia and north-eastern Thailand. *Pfpm2* amplification was only observed in parasites with the C580Y ‘long haplotype’
*Pfexo* E415G mutation	NCT02282293 Reducing the Burden of Malaria in HIV-Infected Pregnant Women and Their HIV-Exposed Children (PROMOTE Birth Cohort 2)	Randomizedcontrolled trial among 300 pregnant women in Uganda. Participants were randomizedd to receive either: (i) SP (500 mg Sulphadoxine and 25 mg pyrimethamine) every 8 weeks, (ii) DHA-PPQ (40 mg dihydroartemisinin and 320 mg PQ) every 8 weeks, or (iii) DHA-PPQ every 4 weeks	Conrad et al. (2017) [53] Impact of Intermittent Preventive Treatment During Pregnancy on *Plasmodium falciparum* Drug Resistance–Mediating Polymorphisms in Uganda	Conrad et al.sequenced a 395 bp amplicon surrounding the E415G locus but did not find the E415G mutation in the samples that they analysed. They detected a different non-synonymous mutation in *pfexo,* R346T, in 1 of 19 samples collected from patients receiving DHA-PPQ treatment
	NCT02788864 A Randomized, Placebo-controlled, Double-blind Trial Using Dihydroartemisinin and Piperaquine (DHA-PPQ) to Protect Forest Workers From Malaria in Bu Gia Map National Park	Active comparator: DHA-PPQ for 3 days prior to forest visit Placebo comparator: Placebo for 3 days prior to forest visit 150 participants	Son et al. (2017) [58] The prevalence, incidence and prevention of *Plasmodium falciparum* infections in forest rangers in Bu Gia Map National Park, Binh Phuoc province, Vietnam: a pilot study	This study found that before MDA, 11 out of 30 samples had the E415G mutation. Following MDA and returning from working in the forest, two study participants were infected with *P. falciparum.* One in the DHA-PPQ arm and one in the placebo arm. The participant in the placebo arm was infected with wild-type *P. falciparum* when they entered the forest, but the E415G mutation when they returned
*Pfcrt* mutations	NCT02793622 Prevention of Malaria in HIV-uninfected Pregnant Women and Infants	IPTp during pregnancy: Active comparator arm: Monthly SP during pregnancy Active comparator arm: Monthly DHA-PPQ during pregnancy 782 participants	Nayebare et al. (2020) [59] Associations between Malaria-Preventive Regimens and *Plasmodium falciparum* Drug Resistance-Mediating Polymorphisms in Ugandan Pregnant Women	This study found that *pfcrt* K76**T** was more prevalent in parasites collected while women received IPTp with DHA-PPQ, than in parasites collected before the start of IPTp, or while women received IPTp with SP
	Samples from multiple trials and epidemiological studies conducted between 1st Jan 2007 and 31st December 2018 were analysed by Imwong et al. (2020). Therapeutic efficacy studies of artesunate [50] and DHA-PPQ in Yunnan Province of China, from 2009 to 2012	Multiple trials and epidemiological studies, including MDA with DHA-PPQ in Kayin State, Myanmar	Imwong et al. (2020) [51] Molecular epidemiology of resistance to antimalarial drugs in the Greater Mekong subregion: an observational study	This study found no evidence for selection of *pfcrt* mutations associated with piperaquine resistance following MDA with DHA-PPQ in Kayin State, Myanmar, or in Cambodia
	NCT02914145 Mass Drug Administration of Monthly DHA-PQP to Accelerate Towards Malaria Elimination in Magude District, Southern Mozambique	Mass Drug Administration with DHA-PPQ. Two monthly rounds of MDA with DHA-PPQ for two consecutive years in Magude, Southern Mozambique	Gupta et al. (2020) [52] Effect of mass dihydroartemisinin–piperaquine administration in southern Mozambique on the carriage of molecular markers of antimalarial resistance	This study found no statistically significant difference between the frequency of *pfcrt* polymorphisms when comparing samples collected before and after MDA
	NCT02282293 Reducing the Burden of Malaria in HIV-Infected Pregnant Women and Their HIV-Exposed Children (PROMOTE Birth Cohort 2)	Randomized controlled trial among 300 pregnant women in Uganda. Participants were randomizedto receive either: (i) SP (500 mg Sulphadoxine and 25 mg pyrimethamine) every 8 weeks, (ii) DHA-PPQ (40 mg dihydroartemisinin and 320 mg PQ) every 8 weeks, or (iii) DHA-PPQ every 4 weeks	Wallender et al. (2019) [60] Modelling Prevention of Malaria and Selection of Drug Resistance with Different Dosing Schedules Dihydroartemisinin-Piperaquine Preventive Therapy during Pregnancy in Uganda	This study used non-linear mixed effects modelling to describe the relationship between PPQ concentration and the probability of finding the mutation N86Y in *pfmdr1* and K76T in *pfcrt*. The models predicted that higher median PPQ concentrations would be required to prevent infections with mutant haplotypes compared to wild-type haplotypes
			Conrad et al. (2017) [53] Impact of Intermittent Preventive Treatment During Pregnancy on *Plasmodium falciparum* Drug Resistance–Mediating Polymorphisms in Uganda	Treatment with DHA-PPQ was associated with increased prevalence of *pfcrt* K76**T** compared with samples collected before treatment, or treatment with SP. Selection increased with increasing PPQ exposure. Receipt of DHA-PPQ selected for the *pfcrt* 76T-*mdr1* N86-184F-1246D haplotype
	NCT00527800 Interactions Between HIV and Malaria in African Children 5 year longitudinal trial from 2007 to 2012 in Tororo, Uganda	Experimental arm 1: Treatment for episodes of uncomplicated malaria with DHA-PPQ, once daily for 3 days Comparator arm 2: Treatment for uncomplicated malaria with artemether-lumefantrine, twice daily for 3 days Experimental A: Prevention of malaria in HIV uninfected, exposed children, with trimethoprim-sulfamethoxazole No intervention B: Prevention of malaria in HIV uninfected, exposed children	Conrad et al. (2014) [42] Comparative Impacts Over 5 Years of Artemisinin-Based Combination Therapies on *Plasmodium falciparum* Polymorphisms That Modulate Drug Sensitivity in Ugandan Children	Treatment over time with DHA-PPQ or AL was associated with higher prevalences of wildtype K76. The extent of selection was lower than that with recent treatment with AL
	NCT00941785 Randomized Trial of the Efficacy, Safety, Tolerability and Pharmacokinetics of Dihydroartemisinin-piperaquine for Seasonal IPT to Prevent Malaria in Children Under 5 Years	Experimental: Three monthly rounds of DHA-PPQ in August, September and October Active comparator: Three monthly rounds of Sulphadoxine-Pyrimethamine plus Amodiaquine 1500 particpants	Zongo et al. (2015) [61] Randomized Noninferiority Trial of Dihydroartemisinin-Piperaquine Compared with Sulphadoxine-Pyrimethamine plus Amodiaquine for Seasonal Malaria Chemoprevention in Burkina Faso	This study found no significant difference in prevalence of the *pfcrt* mutation K76T following SMC with DHA-PPQ
			Somé et al. (2014) [62] Selection of Drug Resistance-Mediating *Plasmodium falciparum* Genetic Polymorphisms by Seasonal Malaria Chemoprevention in Burkina Faso	This study measured the prevalence of mutations in parasites collected before the initiation of the intervention, a month after the completion of three-monthly treatments, and from a control group of children not subject to the intervention. They found no significant selection of *pfcrt* K76**T** following treatment with DHA-PPQ
	NCT00948896 and NCT00978068	Experimental arm 1: TS; TMP/SMX given daily Experimental arm 2: SP given monthly as a single dose Experimental arm 3: DHA-PPQ given monthly, once a day for 3 consecutive days Arm 4, no intervention	Tumwebaze et al. (2015) [63] Impact of antimalarial treatment and chemoprevention on the drug sensitivity of malaria parasites isolated from Ugandan children	This study found minor differences in the prevalence of SNPs associated with drug resistance between different trial arms. Monthly DHA-PPQ treatment was not associated with polymorphisms in *pfcrt*
*Pfmdr1* polymorphisms	NCT02793622 Prevention of Malaria in HIV-uninfected Pregnant Women and Infants	IPTp during pregnancy: Active comparator arm: Monthly SP during pregnancy Active comparator arm: Monthly DHA-PPQ during pregnancy 782 participants	Nayebare et al. (2020) [59] Associations between Malaria-Preventive Regimens and *Plasmodium falciparum* Drug Resistance-Mediating Polymorphisms in Ugandan Pregnant Women	This study found that *pfmdr1* N86**Y** and Y184**F** were more prevalent in parasites collected while women received IPTp with DHA-PPQ, than in parasites collected before the start of IPTp, or while women received IPTp with SP. The prevalence of D1246**Y** was similar in parasites collected before and after IPTp
	NCT02914145 Mass Drug Administration of Monthly DHA-PQP to Accelerate Towards Malaria Elimination in Magude District, Southern Mozambique	Mass Drug Administration with DHA-PPQ. Two monthly rounds of MDA with DHA-PPQ for two consecutive years in Magude, Southern Mozambique	Gupta et al. (2020) [52] Effect of mass dihydroartemisinin–piperaquine administration in southern Mozambique on the carriage of molecular markers of antimalarial resistance	This study found no evidence of statistically significant differences in *pfmdr1* polymorphisms, including *pfmdr1* copy number, when comparing samples collected before and after MDA
	Samples from multiple trials and epidemiological studies conducted between 1st Jan 2007 and 31st December 2018 were analysed by Imwong et al. (2020). Therapeutic efficacy studies of artesunate [50] and DHA-PPQ in Yunnan Province of China, from 2009 to 2012	Multiple trials and epidemiological studies, including MDA with DHA-PPQ in Kayin State, Myanmar	Imwong et al. (2020) [51] Molecular epidemiology of resistance to antimalarial drugs in the Greater Mekong subregion: an observational study	This study found that *pfmdr1* amplification is at low prevalence across the Greater Mekong subregion
	NCT02083380 Randomized Phase IIb Study of Efficacy, Safety, Tolerability & Pharmacokinetics of a Single Dose Regimen of Artefenomel (OZ439) in Loose Combination With Piperaquine in Adults and Children With Uncomplicated *Plasmodium falciparum* Malaria	Experimental: A) Artefenomel 800 mg and Piperaquine Phosphate 640 mg Experimental: B) Artefenomel 800 mg and Piperaquine Phosphate 960 mg Experimental: C) Artefenomel 800 mg and Piperaquine Phosphate 1440 mg	Leroy et al. (2019) [56] African isolates show a high proportion of multiple copies of the *Plasmodium falciparum* plasmepsin-2 gene, a piperaquine resistance marker	This study analysed *pfmdr1* copy number in samples collected from patients before treatment. They found a threefold higher prevalence of multicopy *pfmdr1* in Africa than in Asia. Parasites with multicopy *pfpm2* and single copy *pfmdr1* (which is hypothesized to favour PPQ resistance) were found at similar prevalence in Asian and African samples
	NCT02282293 Reducing the Burden of Malaria in HIV-Infected Pregnant Women and Their HIV-Exposed Children (PROMOTE Birth Cohort 2)	Randomized controlled trial among 300 pregnant women in Uganda. Participants were randomized to receive either: (i) SP (500 mg Sulphadoxine and 25 mg pyrimethamine) every 8 weeks, (ii) DHA-PPQ (40 mg dihydroartemisinin and 320 mg PQ) every 8 weeks, or (iii) DHA-PPQ every 4 weeks	Wallender et al. (2019) [60] Modelling Prevention of Malaria and Selection of Drug Resistance with Different Dosing Schedules Dihydroartemisinin-Piperaquine Preventive Therapy during Pregnancy in Uganda	This study used non-linear mixed effects modelling to describe the relationship between PPQ concentration and the probability of finding the mutation N86Y in *pfmdr1* and K76T in *pfcrt*. The models predicted that higher median PPQ concentrations would be required to prevent infections with mutant haplotypes compared to wild-type haplotypes
			Conrad et al. (2017) [53] Impact of Intermittent Preventive Treatment During Pregnancy on *Plasmodium falciparum* Drug Resistance–Mediating Polymorphisms in Uganda	Treatment with DHA-PPQ was associated with increased prevalence of *pfmdr1* N86**Y** and Y184**F** mutations compared with before treatment, or treatment with SP. Increased frequency of DHA-PPQ exposure was associated with increased prevalence of *pfmdr1* N86**Y**. Treatment with DHA-PPQ was associated with decreased prevalence of D1246**Y** compared with samples collected before treatment, or treatment with SP. DHA-PPQ treatment selected for the *pfmdr1* 86**Y**-184**F**, 86**Y**-**D**1246, **N**86-184**F**-1246**D** and the *pfcrt* 76**T**-*mdr1* **N**86-184**F**-1246**D** haplotypes. Consistent with this, a generalized linear model of PPQ exposure demonstrated that increasing PPQ concentration was associated with increasing prevalence of N86**Y**. There was no evidence of increased *pfmdr1* copy number associated with DHA-PPQ treatment
	NCT02788864 A Randomized, Placebo-controlled, Double-blind Trial Using Dihydroartemisinin and Piperaquine (DHA-PPQ) to Protect Forest Workers From Malaria in Bu Gia Map National Park	Active comparator: DHA-PPQ for 3 days prior to forest visit Placebo comparator: Placebo for 3 days prior to forest visit 150 participants	Son et al. (2017) [58] The prevalence, incidence and prevention of *Plasmodium falciparum* infections in forest rangers in Bu Gia Map National Park, Binh Phuoc province, Vietnam: a pilot study	This study found that before MDA, no *P. falciparum* isolates that were genotyped had multiple copies of *pfmdr1.* Following MDA and returning from working in the forest, two study participants were infected with *P. falciparum.* Neither had multicopy *pfmdr1*
	NCT00941785 Randomized Trial of the Efficacy, Safety, Tolerability and Pharmacokinetics of Dihydroartemisinin-piperaquine for Seasonal IPT to Prevent Malaria in Children Under 5 Years	Experimental: Three monthly rounds of DHA-PPQ in August, September and October Active comparator: Three monthly rounds of Sulphadoxine-Pyrimethamine plus Amodiaquine 1500 participants	Zongo et al. (2015) [61] Randomized Noninferiority Trial of Dihydroartemisinin-Piperaquine Compared with Sulphadoxine-Pyrimethamine plus Amodiaquine for Seasonal Malaria Chemoprevention in Burkina Faso	This study found no significant difference in the prevalence of *pfmdr1* mutations N86Y, F184Y or D1246Y following SMC with DHA-PPQ
			Somé et al. (2014) [62] Selection of Drug Resistance-Mediating *Plasmodium falciparum* Genetic Polymorphisms by Seasonal Malaria Chemoprevention in Burkina Faso	Measured the prevalence of mutations in parasites collected from children before the initiation of the intervention, from children a month after the completion of three-monthly treatments, and from a control group of children not subject to the intervention. They found borderline significant selection for *pfmdr1* **D**1246Y following treatment with DHA-PPQ
	NCT00527800 Interactions Between HIV and Malaria in African Children 5 year longitudinal trial from 2007 to 2012 in Tororo, Uganda	Experimental arm 1: Treatment for episodes of uncomplicated malaria with DHA-PPQ, once daily for 3 days Comparator arm 2: Treatment for uncomplicated malaria with artemether-lumefantrine, twice daily for 3 days Experimental A: Prevention of malaria in HIV uninfected, exposed children, with trimethoprim-sulfamethoxazole No intervention B: Prevention of malaria in HIV uninfected, exposed children	Taylor et al. (2016) [64] Artemether-Lumefantrine and Dihydroartemisinin-Piperaquine Exert Inverse Selective Pressure on *Plasmodium falciparum* Drug Sensitivity-Associated Haplotypes in Uganda	This study fit a haplotype frequency estimation model to study *pfmdr1* N86Y, Y184F and D1246Y alleles. They found that DHA-PPQ selected for 86**Y**, but only when this was combined with **Y**184 and 1246**Y**, selecting for the haplotype YYY, and against haplotypes NFD and NYY
			Conrad et al. (2014) [42] Comparative Impacts Over 5 Years of Artemisinin-Based Combination Therapies on *Plasmodium falciparum* Polymorphisms That Modulate Drug Sensitivity in Ugandan Children	The prevalences of N86, 184F and D1246 increased over the time of the study. When comparing the AL and DHA-PPQ treatment arms, the prevalences of these alleles was greater in the AL treatment arm. Recent treatment with DHA-PPQ was associated with increased prevalence of *pfmdr1* N86**Y**, D1246**Y** and a lower prevalence of Y184**F**. The extent of selection was lower than that with recent treatment with AL. There was no association between treatment arm and *pfmdr1* copy number
	NCT00948896 A Randomized Controlled Trial of Monthly Dihydroartemisinin-piperaquine Versus Monthly Sulfadoxine-pyrimethamine Versus Daily Trimethoprim-sulfamethoxazole Versus No Therapy for the Prevention of Malaria	Experimental arm 1: TS; TMP/SMX given daily Experimental arm 2: SP given monthly as a single dose Experimental arm 3: DHA-PPQ given monthly, once a day for 3 consecutive days Arm 4, no intervention	Tumwebaze et al. (2015) [63] Impact of antimalarial treatment and chemoprevention on the drug sensitivity of malaria parasites isolated from Ugandan children	This study found minor differences in the prevalence of SNPs associated with drug resistance between different trial arms. Monthly DHA-PPQ treatment was not associated with polymorphisms in *pfmdr1*. However, when samples from the DHA-PPQ treatment arm were sorted based on circulating PPQ levels, parasites with higher PPQ exposure showed evidence for selection for *pfmdr1* 86**Y** and 1246**Y** mutations
			Ochong et al. (2013) [65] Fitness Consequences of *Plasmodium falciparum pfmdr1*Polymorphisms Inferred from Ex Vivo Culture of Ugandan Parasites	This study did not measure changes in molecular markers of resistance in trial participants; this study cultured parasites from participants to evaluate fitness advantages of certain *pfmdr1* polymorphisms. Their results suggest fitness advantages for parasites with the *pfmdr1* N86**Y** mutation and wild-type **D**1246Y
*Artemisinin-resistance associated mutations*	NCT02914145 Mass Drug Administration of Monthly DHA-PQP to Accelerate Towards Malaria Elimination in Magude District, Southern Mozambique	Mass Drug Administration with DHA-PPQ. Two monthly rounds of MDA with DHA-PPQ for two consecutive years in Magude, Southern Mozambique	Gupta et al. (2020) [52] Effect of mass dihydroartemisinin–piperaquine administration in southern Mozambique on the carriage of molecular markers of antimalarial resistance	There was no statistically significant difference between the frequency of polymorphisms when comparing samples collected pre and post MDA. No *pfkelch13* polymorphisms associated with partial-resistance to artemisinin were found in the isolates analyzed
	Samples from multiple trials and epidemiological studies conducted between 1st Jan 2007 and 31st December 2018 were analysed by Imwong et al. (2020). Therapeutic efficacy studies of artesunate[50] and DHA-PPQ in Yunnan Province of China, from 2009 to 2012	Multiple trials and epidemiological studies, including MDA with DHA-PPQ in Kayin State, Myanmar	Imwong et al. (2020) [51] Molecular epidemiology of resistance to antimalarial drugs in the Greater Mekong subregion: an observational study	The longitudinal study found that two genotypes of *pfkelch* have come to dominate: the Cys580Tyr mutation in the eastern Greater Mekong subregion, and the Phe446Ile mutation in Myanmar. They found no evidence for further selection of *pfkelch* mutations following mass treatment with DHA-PPQ
	NCT01872702 Targeted Chemo-elimination (TCE) to Eradicate Malaria in Areas of Suspected or Proven Artemisinin Resistance in Southeast Asia and South Asia	Active comparator: Three monthly rounds of DHA-PPQ and low dose primaquine Placebo comparator: No intervention used Two villages randomly allocated to intervention at each of 4 sites, and two villages randomly allocated to control (no intervention). 500 people per village	von Seidlein et al. (2019) [54] The impact of targeted malaria elimination with mass drug administrations on *falciparum* malaria in Southeast Asia: A cluster randomized trial	This study analysed blood specimens from before MDA initiation. They found that 4% of the individuals sampled (10 of 269) had *pfkelch13* C580Y and multiple copies of *pfpm2/3.* This study did not measure the difference in prevalence of markers pre and post MDA. 9/10 of the participants with the C580Y genotyped parasites cleared their parasitaemia after receiving the MDA
			Tripura et al. (2018) [66] A Controlled Trial of Mass Drug Administration to Interrupt Transmission of Multidrug-Resistant Falciparum Malaria in Cambodian Villages	This study found that all genotyped *Plasmodium* infections carried the *pfkelch13* C580Y mutation. Of isolates genotyped up to April 2016, 34 had the C580Y mutation and 1 had the F446I mutation
			Landier et al. (2017) [55] Safety and effectiveness of mass drug administration to accelerate elimination of artemisinin-resistant falciparum malaria: A pilot trial in four villages of Eastern Myanmar	This study measured the prevalence of *pfkelch13* mutations before and after MDA. They found that the prevalence was 85.6% before and 56.7% afterwards. The most frequent mutations were C580Y and G358V
	NCT number not available Large-scale Artemisinin-Piperaquine Mass Drug Administration with or Without Primaquine, Anjouan Island, Union of Comoros	Artemisinin-piperaquine (AP), with or without primaquine, was given in 3 monthly rounds as MDA	Deng et al. (2018) [67] Large-scale Artemisinin–Piperaquine Mass Drug Administration With or Without Primaquine Dramatically Reduces Malaria in a Highly Endemic Region of Africa	This study found no evidence for the selection of *pfkelch* mutations in the analysis of 52 malaria samples collected following MDA with artemisinin-piperaquine (with or without PMQ)
	NCT02282293 Reducing the Burden of Malaria in HIV-Infected Pregnant Women and Their HIV-Exposed Children (PROMOTE Birth Cohort 2)	Randomized controlled trial among 300 pregnant women in Uganda. Participants were randomized to receive either (i) SP (500 mg Sulphadoxine and 25 mg pyrimethamine) every 8 weeks (ii) DHA-PPQ (40 mg dihydroartemisinin and 320 mg PQ) every 8 weeks or (iii) DHA-PPQ every 4 weeks	Conrad et al. (2017) [53] Impact of Intermittent Preventive Treatment During Pregnancy on *Plasmodium falciparum* Drug Resistance-Mediating Polymorphisms in Uganda	This study found no evidence for the selection of *pfkelch13* mutations between treatment groups
	NCT02083380 Randomized Phase IIb Study of Efficacy, Safety, Tolerability & Pharmacokinetics of a Single Dose Regimen of Artefenomel (OZ439) in Loose Combination With Piperaquine in Adults and Children With Uncomplicated *Plasmodium falciparum* Malaria	Experimental: A) Artefenomel 800 mg and Piperaquine Phosphate 640 mg Experimental: B) Artefenomel 800 mg and Piperaquine Phosphate 960 mg Experimental: C) Artefenomel 800 mg and Piperaquine Phosphate 1440 mg	Leroy et al. (2019) [56] African isolates show a high proportion of multiple copies of the *Plasmodium falciparum* plasmepsin-2 gene, a piperaquine resistance marker	This study analysed samples collected from patients before treatment. They found that 67.6% of isolates genotyped from Vietnam had *pfkelch13* resistance mutations. In contrast, none of the 332 isolates successfully genotyped from African patients carried validated or candidate *pfkelch13* mutations
			Macintyre et al. (2017) [68] A randomised, double-blind clinical phase II trial of the efficacy, safety, tolerability and pharmacokinetics of a single dose combination treatment with artefenomel and piperaquine in adults and children with uncomplicated *Plasmodium falciparum* malaria	This study analysed *pfkelch13* mutations and found a high mutation frequency in Vietnam of 70.1%. Five mutations were detected, including C580Y, I543T, P553L and V568G, all associated with artemisinin partial resistance, and C469P, which is not associated with artemisinin partial resistance. The presence of these mutations was associated with parasite clearance half-life. The most common *pfkelch13* genotypes were C580Y and P553L
	Samples from multiple trials and epidemiological studies were analysed by Imwong et al. (2017). Those that were trials that included PPQ or DHA-PPQ were NCT02453308	Longitudinal observational study between 1st January 2008 and 31st December 2015	Imwong et al. (2017) [57] The spread of artemisinin-resistant *Plasmodium falciparum* in the Greater Mekong subregion: a molecular epidemiology observational study	This study found that over a large area of the Greater Mekong subregion, a single long haplotype *pfkelch* C580Y mutant lineage has come to dominate
	NCT02788864 A Randomized, Placebo-controlled, Double-blind Trial Using Dihydroartemisinin and Piperaquine (DHA-PPQ) to Protect Forest Workers From Malaria in Bu Gia Map National Park	Active comparator: DHA-PPQ for 3 days prior to forest visit Placebo comparator: Placebo for 3 days prior to forest visit 150 participants	Son et al. (2017) [58] The prevalence, incidence and prevention of *Plasmodium falciparum* infections in forest rangers in Bu Gia Map National Park, Binh Phuoc province, Vietnam: a pilot study	This study found that before MDA, 11 out of 30 *P. falciparum* had the C580Y *kelch13* mutation. Following MDA and returning from working in the forest, two study participants were infected with *P. falciparum.* One in the DHA-PPQ arm and one in the placebo arm. The participant in the placebo arm was infected with wild-type *P. falciparum* when they entered the forest, but the C580Y mutation when they returned

## Discussion

Advances in sequencing technology have resulted in an explosion in the generation, availability, and analysis of sequencing data. This includes genomic data from the deadliest malaria parasite, *P. falciparum.* Genomic surveillance has consequently gained an increasingly important role in monitoring anti-malarial drug resistance, through the surveillance of molecular markers in the *P. falciparum* genome. The surveillance of molecular markers associated with drug resistance is recognized as a surveillance tool by the WHO [47]. Genomic surveillance is particularly important in the case of mass drug administration programmes, where drug treatment is given to members of a population whether or not they are symptomatic for malaria. MDA is endorsed by the WHO in certain settings, such as endemic island communities, where there is limited risk of importation of infection, good access to treatment and implementation of vector control and surveillance [2]. Furthermore, randomized controlled trials with MDA using DHA-PPQ have been shown to be safe and to significantly lower the burden of malaria in pre-elimination settings [48]. Therefore, with continued use of MDA, surveillance of molecular markers of resistance is crucial.

## What impact did these studies have on molecular markers of drug resistance?

### Pfpm2 copy number

There was no evidence for selection for increased *pfpm2* copy number following MDA with DHA-PPQ in Kayin state, Myanmar [51] or after MDA for 2 months over 2 consecutive years in Mozambique [52] or after MDA taken for 3 days for 3 months in Myanmar [55]. Conrad et al. found modest increases in *pfpm2* copy number in 1 of 18 samples from patients receiving DHA-PPQ IPTp, where participants received DHA-PPQ every 8 weeks or every 4 weeks during pregnancy [53]. Taken together, this suggests that short term MDA treatments are unlikely to select for amplification in *pfpm2* copy number. However, Imwong et al. have found *pfpm2* amplification in their longitudinal observational studies in the eastern Greater Mekong subregion [51, 57]. This is an area where DHA-PPQ has been used extensively for many years, and may suggest that longer periods of DHA-PPQ use can select for increased *pfpm2* copy number.

### Pfexo E415G

Two studies included in this review measured the frequency of the *pfexo* E415**G** mutation. Conrad et al. sequenced this locus and did not detect the *pfexo* E415**G** mutation in the samples that they analysed [53]. Son et al. identified the *pfexo* E415**G** mutation in their study population prior to MDA, but found no statistically significant increase in the prevalence of this mutation following MDA with forest rangers in Vietnam [58]. This evidence does not demonstrate a correlation between MDA with DHA-PPQ and increased prevalence of the *pfexo* E415**G** mutation. However, only 2 of the studies analysed this marker. Of note, some of the 20 studies analysed in this review were published before the association between the *pfexo* mutation and PPQ resistance was reported. This includes Ochong et al., Conrad et al., Somé et al., Zongo et al., Tumwebaze et al., Taylor et al. and Madanitsa et al. [42, 49, 61–65].

### Pfcrt mutations

Nayebare et al. found that *pfcrt* K76**T** prevalence was higher in samples collected from women in Uganda during IPTp with DHA-PPQ than in parasites collected prior to the start of IPTp, or while women received IPTp with SP [59]. Similarly, Conrad et al. found that the prevalence of the K76**T** mutation was higher in samples collected from the DHA-PPQ arm of IPTp in Uganda, than in the SP arm or in samples collected prior to the start of IPTp [53]. This increase in K76**T** prevalence was also correlated with increased PPQ exposure.

In contrast, Somé et al. found no significant selection for *pfcrt* K76**T** following Seasonal Malaria Chemoprevention (SMC) with DHA-PPQ for 3 months in Burkina Faso [62]. Imwong et al. analysed *pfcrt* mutations F145**I**, I218**F**, N326**S**, M343**I/L** and G353**V** and found no evidence of selection of *pfcrt* mutations associated with PPQ resistance following MDA in Kayin State, Myanmar [51]. In support of this, Gupta et al. found no statistically significant difference in the prevalence of *pfcrt* polymorphisms after MDA with DHA-PPQ for 2 months, for 2 years, in Mozambique [52]. Tumwebaze et al. found that monthly MDA with DHA-PPQ in Uganda was not associated with changes in the prevalence of *pfcrt* polymorphisms [63]. Finally, Zongo et al. found no significant difference in the prevalence of the K76**T** polymorphism following SMC with DHA-PPQ in Burkina Faso [61].

Overall, evidence for selection of *pfcrt* K76**T** following MDA with DHA-PPQ is mixed. Few studies analysed *pfcrt* markers other than K76**T**. Other key polymorphisms have been associated with PPQ resistance in vitro. *Pfcrt* H97**Y**, F145**I**, M343**L**, G353**V** [21] and C101**F** [22] mutations have been shown to confer PPQ resistance and CQ sensitivity in vitro*,* and F145**I** and C350**R** have been shown to efflux PPQ from the DV at the same time as reducing CQ transport [24]. Furthermore, T93**S** and I218**P** have been shown to confer PPQ resistance [35] along with T93**S**, H97**Y**, F145**I** and I128**F**. Each of these mutations conferred resistance to PPQ on a background of *pfexo* E145**G** [36]. This evidence suggests that that there may be other, more relevant *pfcrt* markers than K76**T**, and that future studies would benefit from monitoring this range of identified putative *pfcrt* polymorphisms which have conferred DHA-PPQ resistance in vitro.

### Pfmdr1 mutations and copy number

Following IPTp with DHA-PPQ in Uganda, both Nayebare et al. and Conrad et al. found increased prevalence of *pfmdr1* N86**Y** and Y184**F** mutations in samples collected during treatment than in samples collected before treatment [53, 59]. Increased exposure to PPQ also correlated with increased prevalence of N86**Y** [53, 63] and D1246**Y** [63] in Uganda. Mixed results were found regarding the D1246**Y** polymorphism. Nayebare et al. found that the prevalence of *pfmdr1* D1246**Y** was similar in samples collected before and after IPTp [59]. Whereas, Conrad et al. found that D1246**Y** prevalence decreased in samples collected during IPTp with DHA-PPQ, compared with samples collected before treatment or during treatment with SP [53]. Somé et al. found borderline selection for wild-type **D**1246Y following treatment with DHA-PPQ in Burkina Faso [62]. Taylor et al. and Conrad et al. monitored changes in polymorphisms and haplotypes in *pfmdr1* in Uganda between 2007 and 2012 after treatment with AL or DHA-PPQ [42, 64]. Conrad et al. found that treatment with DHA-PPQ was associated with increased prevalence of N86**Y** and D1246**Y**, and a lower prevalence of Y184**F** [42]. Taylor et al. used a haplotype frequency estimation model and found that treatment with DHA-PPQ only selected for N86**Y** when this allele was found with D1246**Y** and **Y**184F, in the haplotype YYY, and that it selected against haplotypes NFD and NYY [64].

In contrast, Gupta et al. found no change in *pfmdr1* polymorphisms following MDA with DHA-PPQ in Mozambique [52]. Furthermore, Zongo et al. found no significant difference in the prevalence of *pmdfr1* mutations following SMC with DHA-PPQ in Burkina Faso [61]. Gupta et al., Son et al., and Conrad et al., found no association between MDA with DHA-PPQ and increased *pfmdr1* copy number [42, 52, 53, 58]. This small number of studies suggests that MDA with DHA-PPQ may select for mutations in *pfmdr1,* particularly N86**Y**.

### Artemisinin partial resistance associated mutations

No evidence for increased selection of *pfkelch13* mutations was found after MDA with DHA-PPQ in Kayin State, Myanmar [51] in Mozambique [52], Uganda [53], or in Eastern Myanmar [55], or following treatment with artemisinin-piperaquine in the Comoros [67]. This suggests that although the prevalence of some *pfkelch13* polymorphisms have become widespread in the GMS [51, 57], MDA with DHA-PPQ has not increased the prevalence of *pfkelch13* mutations associated with reduced artemisinin sensitivity, as least in the studies which have monitored molecular markers of resistance following MDA.

## Conclusion

Despite molecular markers of drug resistance being a recognized surveillance tool by the WHO [27], the level of reporting of molecular markers associated with DHA-PPQ resistance found in this study was low. Of the total 96 papers screened for eligibility in this review, only 20 analysed molecular markers of drug resistance. This highlights considerations for future studies with DHA-PPQ, where further analysis and reporting of molecular markers related to DHA-PPQ resistance would greatly assist understanding of how MDA impacts polymorphisms associated with resistance. Importantly, molecular markers associated with DHA-PPQ have different prevalences in different geographic regions. Molecular surveillance data from future studies in different geographies will further increase understanding of how treatment with DHA-PPQ is impacting the evolution of resistance in these different geographies.

The choice of markers analysed is not currently standardized, which may be partly because *pfexo* E415G and *pfpm2* copy number have only recently emerged as molecular markers associated with PPQ resistance. Future studies with DHA-PPQ should monitor a broader range of molecular markers which have been associated with resistance to DHA-PPQ. This includes *pfexo* E415G*, pfpm2* copy number and *pfmdr1* copy number and N86Y, Y184F and D1246Y mutations. In addition to these markers, putative polymorphisms in *pfcrt* should also be monitored, including mutations T93S, H97Y, F145I, I218P, M343L, C350R, G353V. This would contribute to a more comprehensive analysis of resistance polymorphisms following MDA implementation.

To really understand the impact that DHA-PPQ MDA has had on the evolution of drug resistance, there needs to be much greater focus and investment on genomic surveillance in trial and programmatic settings. This would enable the research community to build on the already growing field of genomic surveillance to better understand the impact of using anti-malarial drugs on a large scale. Phenomenal steps have already been made, including through the Pan-African Malaria Genetic Epidemiology Network (PAMGEN). However, the lack of published molecular surveillance data from trials highlights the need for increasing focus on genomic surveillance if MDA is used as a population-based strategy for malaria control.

## Data Availability

The processed datasets are available from the corresponding author. All data is available from the individual studies cited, including ENA accession numbers for raw sequences.

## Detailed search methodology

Detailed search terms used in MEDLINE

Ovid MEDLINE(R) ALL < 1946 to May 24, 2021 > 

1. Malaria/or malaria, cerebral/or malaria, falciparum/or blackwater fever/or malaria, vivax/67560
2. (Malaria* or falciparum or plasmodium infection* or remittent fever or blackwater fever).mp. 107016
3. 1 or 2 107016
4. Chemoprevention/or mass drug administration/6658
5. Mass screening/or anonymous testing/or multiphasic screening/108880
6. [Mass adj3 (screening* or administration* or test* or treatment*)].mp.121669
7. (MSAT or MTAT or FSAT or FEMSE or MPPT or TME or DOT or RACD or IPTp or SMC).mp.52228
8. Direct observed therap*.mp.27
9. Mass piperaquine prophylactic treat*.mp.0
10. Seasonal malaria chemo*.mp.115
11. (Focal screening and treat*).mp.6
12. Intermittent preventive treatment in pregnancy.mp.148
13. Reactive case detection.mp.71
14. (Focal screening and treatment).mp.6
15. 4 or 5 or 6 or 7 or 8 or 9 or 10 or 11 or 12 or 13 or 14 181089
16. 3 and 15 2086
17. Exp Antimalarials/85757
18. (Anti-malarial* or antimalarial*).mp.35587
19. Drug resistance/or drug resistance, multiple/or drug tolerance/83191
20. [(Drug or multidrug) adj3 (resist* or toleran* or tolerate* or sensit*)].mp.368770
21. [(Drug or multidrug) adj3 (resist* or toleran* or tolerate* or sensit*)].mp.368770
22. 17 or 18 or 19 or 20 or 21 448688
23. 16 and 22 1195
24. (Piperaquine or dihydroartemisinin or dihydroartemisinin-piperaquine).mp. [mp = title, abstract, original title, name of substance word, subject heading word, floating sub-heading word, keyword heading word, organism supplementary concept word, protocol supplementary concept word, rare disease supplementary concept word, unique identifier, synonyms] 2129
25. 23 and 24 117

These studies were filtered to included publication dates of 2011 onwards, resulting in 99 papers in total. Detailed search terms used in EMBASE. Embase <1974 to 2021 May 24>

1. Malaria falciparum/or *Plasmodium vivax* malaria/ or malaria/ or *Plasmodium knowlesi* malaria/ or *Plasmodium ovale* malaria/88321
2. (Malaria* or falciparum or plasmodium infection* or remittent fever or blackwater fever).mp.130248
3. 1 or 2 130248
4. Mass drug administration/or chemoprophylaxis/26856
5. Mass screening/56612
6. [Mass adj3 (screening* or administration* or test* or treatment*)].mp.76289
7. Direct observed therap*.mp.65
8. Mass piperaquine prophylactic treat*.mp.0
9. Seasonal malaria chemo*.mp.255
10. (Focal screening and treat*).mp.16
11. Intermittent preventive treatment in pregnancy.mp.226
12. Reactive case detection.mp.157
13. (Focal screening and treatment).mp.15
14. 4 or 5 or 6 or 7 or 8 or 9 or 10 or 11 or 12 or 13 102462
15. 3 and 14 3684
16. Antimalarial agent/24465
17. (Anti-malarial* or antimalarial*).mp.41035
18. Antimalarial drug resistance/ or antimalarial drug susceptibility/ or drug resistance/ or multidrug resistance/143743
19. [(Drug or multidrug) adj3 (resist* or toleran* or tolerate* or sensit*)].mp.470284
20. 16 or 17 or 18 or 19 502467
21. 15 and 20 1461
22. (Dihydroartemisinin or dihydroartemisinin plus piperaquine or piperaquine).mp. [mp = title, abstract, heading word, drug trade name, original title, device manufacturer, drug manufacturer, device trade name, keyword, floating subheading word, candidate term word] 4363
23. 21 and 22 192

These studies were filtered to included publication dates of 2011 onwards, resulting in 129 papers in total. Removing duplicates resulted in 184 papers. These 134 papers were excluded because they either did not include reference to PPQ or DHA-PPQ, or they did not include reference to drug resistance/efficacy/tolerability or sensitivity in the abstract or title. This resulted in 50 publications which were assessed for eligibility. 35 studies were excluded because they did not include measurement of molecular markers of resistance, leaving 15 studies from MEDLINE or EMBASE which were included in this review.

IDDO search

Search date: 7/7/2021.

In the IDDO database (https://www.iddo.org), the terms ‘Malaria’ and ‘Piperaquine’ were used to identify publications. This resulted in 9 publications, 2 of which included analysis of molecular markers of resistance and were included in this review.

Clinicaltrials.gov search

Search date: 7/7/2021.

The following search (Disease: malaria, Terms: Piperaquine Status: Has results) resulted in 13 clinical studies. Four studies were excluded as these were pharmacokinetic studies of interventions in healthy volunteers. Three studies were excluded as these were studies involving induced blood stage malaria in healthy participants, as opposed to vector transmitted malaria. This resulted in 1 Phase 2 clinical trial and 5 Phase 3 clinical trial remaining. There were 7 publications associated with these clinical trials which included analysis of molecular markers of resistance. Four of these were unique and included in this review.

## Abbreviations

MDA: Mass drug administration
DHA-PPQ: Dihydroartemisinin-piperaquine
WGS: Whole genome sequencing
SNPs: Single nucleotide polymorphisms
WHO: World Health Organization
ACT: Artemisinin-combination therapy
AL: Artemether-lumefantrine
AS-AQ: Artesunate-amodiaquine
ITN: Insecticide-treated nets
IPTp: Intermittent preventive treatment in pregnancy
LLIN: Long-lasting insecticidal nets
SP: Sulfadoxine-pyrimethamine
AS: Artesunate
QN: Quinine
IVM: Ivermectin
DHA: Dihydroartemisinin
DV: Digestive vacuole
PPQ: Piperaquine
CQ: Chloroquine
CNV: Copy number variant
PCR: Polymerase chain reaction
DNA: Deoxyribose nucleic acid
